# The Modification of Circadian Clock Components in Soybean During Domestication and Improvement

**DOI:** 10.3389/fgene.2020.571188

**Published:** 2020-09-30

**Authors:** Man-Wah Li, Hon-Ming Lam

**Affiliations:** ^1^Center for Soybean Research of the State Key Laboratory of Agrobiotechnology and School of Life Sciences, The Chinese University of Hong Kong, Hong Kong, China; ^2^Shenzhen Research Institute, The Chinese University of Hong Kong, Shenzhen, China

**Keywords:** circadian clock, domestication, early flowering 3, Gigantea, pseudo response regulator 3, *J* locus, soybean

## Abstract

Agricultural production is greatly dependent on daylength, which is determined by latitude. Living organisms align their physiology to daylength through the circadian clock, which is made up of input sensors, core and peripheral clock components, and output. The light/dark cycle is the major input signal, moderated by temperature fluctuations and metabolic changes. The core clock in plants functions mainly through a number of transcription feedback loops. It is known that the circadian clock is not essential for survival. However, alterations in the clock components can lead to substantial changes in physiology. Thus, these clock components have become the *de facto* targets of artificial selection for crop improvement during domestication. Soybean was domesticated around 5,000 years ago. Although the circadian clock itself is not of particular interest to soybean breeders, specific alleles of the circadian clock components that affect agronomic traits, such as plant architecture, sensitivity to light/dark cycle, flowering time, maturation time, and yield, are. Consequently, compared to their wild relatives, cultivated soybeans have been bred to be more adaptive and productive at different latitudes and habitats for acreage expansion, even though the selection processes were made without any prior knowledge of the circadian clock. Now with the advances in comparative genomics, known modifications in the circadian clock component genes in cultivated soybean have been found, supporting the hypothesis that modifications of the clock are important for crop improvement. In this review, we will summarize the known modifications in soybean circadian clock components as a result of domestication and improvement. In addition to the well-studied effects on developmental timing, we will also discuss the potential of circadian clock modifications for improving other aspects of soybean productivity.

## Introduction

Studies have shown that plants with circadian clocks synchronized to their environmental conditions gain growth advantage over those not synchronized (Dodd et al., 2005). The circadian clock not only plays roles in growth and development, it is also involved in metabolism and stress responses (Fukushima et al., 2009; Dodd et al., 2015; Nakamichi et al., 2016; Nitschke et al., 2016; Kim et al., 2017; Gil and Park, 2019). Current knowledge of the plant circadian clock mostly came from studies on Arabidopsis, which is a relatively simple model plant. The Arabidopsis circadian clock consists of mainly three transcription feedback loops, namely the central loop, the morning loop, and the evening loop (Staiger et al., 2013; Foo et al., 2016; Gil and Park, 2019). The central loop is made up of a feedback loop made up of circadian clock associated 1/late elongated hypocotyl (CCA1/LHY) and timing of CAB (TOC1; also known as pseudo response regulator 1, PRR1), which are mutual repressors of each other (Alabadi et al., 2001). On the other hand, in the morning loop, CCA1/LHY activates *PRR5*, *PRR7*, and *PRR9*, which in turn inhibit *CCA1*/*LHY* in a negative feedback loop (Mizuno and Nakamichi, 2005). Early flowering 3 (ELF3), early flowering 4 (ELF4), and LUX ARRHYTHMO (LUX) make up the evening complex (EC) that represses *PRRs*, while they themselves are repressed by CCA1/LHY (Huang and Nusinow, 2016). This delicate configuration, together with the input sensors and other peripheral components, allows the clock to oscillate in a cycle of approximately 24 h. Detailed discussions on the Arabidopsis circadian clock could be found in some recent reviews (Staiger et al., 2013; Foo et al., 2016; Shim et al., 2017; Gil and Park, 2019).

Studies on some crop species suggested that domestication has imposed significant changes on the circadian clock, in terms of the phase, amplitude and period (Shor and Green, 2016). Shifting the circadian phase of important biological processes could have big impacts. For example, a 3-bp deletion in the Phytochrome A-associated F-box protein-encoding gene, *EID1* (*Empfindlicher im dunkelroten Licht protein 1*), in the cultivated tomato has led to the lengthening of the clock period (Muller et al., 2016). This transition allowed the day-neutral tomatoes that originated from the equatorial Andean region of South America to adapt to longer daylengths in Mesoamerica and Europe (Muller et al., 2016). The deceleration of the clock reoriented the phase of some biological processes such as photosynthesis with the longer photoperiods in the higher latitudes to maximize the productivity of these processes. Other examples include the *EAM8* from Barley (Faure et al., 2012), a homolog of Arabidopsis *ELF3*, and *Eps3* from wheat (Gawronski et al., 2014), a homolog of Arabidopsis *LUX*. Alleles of these two genes have played important roles in affecting flowering time of the respective crops and altering the phase, period and amplitude of their circadian clocks (Faure et al., 2012; Gawronski et al., 2014). However, the physiological consequences of the altered clock in these two crops as a result of changes in these two genes are still largely unknown.

Up to now, specific studies on soybean circadian rhythm have been limited, but they can be dated back to the 1950s (Bunning, 1954; Brest, 1970). In those days, it has already been discovered that the circadian movement of trifoliate leaves of soybean, as measured with a kymograph, could last for two weeks after shifting to continuous light in a roughly 24-h fashion relying on the internal clock (Brest, 1970). A much more recent study examined the circadian fluctuations of chlorophyll contents using non-destructive multispectral imaging under drought conditions (Pan et al., 2015). Interestingly, circadian rhythm was not only observed in the aerial parts of the soybean plant. Expressing the luciferase reporter gene driven by the soybean *LHY-CCA1-LIKE b2* (*GmLCLb2*) promoter or soybean *PRR9b2* promoter in transgenic soybean hairy roots revealed that the circadian rhythm of the soybean root is out of sync with the clock in the leaf (Wang Y. et al., 2020), suggesting that the circadian clock may play different roles in different organs of the soybean plant. A recent circadian study on soybean observed that the free-running circadian period lengths of elite soybean cultivars are positively correlated to the latitudes associated with the maturity groups that these cultivars belong to (Greenham et al., 2017). Although the study did not involve any wild soybean, since maturity groups are largely the results of domestication and its subsequent diversification (Jiang et al., 2014; Zhou et al., 2015; Wang Y. et al., 2016; Miladinovic et al., 2018), the correlation between period length and latitude/maturity group suggests that alterations in the soybean circadian clock could be a by-product of these processes.

So far, there are a few transcriptomic studies regarding the soybean circadian clock. One study observed that only about 1.8% of the transcriptome from *Glycine max* cv. Williams 82 developing seeds cycled under constant light condition (Hudson, 2010), which was much lower than 6–40% in Arabidopsis seedlings (Covington et al., 2008). Another study identified 3,695 time-indicating genes in the unifoliate leaves of Williams 82, which amount to less than 10% of the total protein-coding genes in the soybean genome (Li M. et al., 2019). The discrepancy between the two species was probably due to organ-specific effects of the clock. However, we cannot rule out the possibility that the oscillation of the clock is normally less robust in cultivated varieties (Shor and Green, 2016), which makes rhythmic genes less likely to be detected.

In addition to the well-known input signals of light and temperature, stresses are also input signals that could greatly alter the clock. It has been found that mild drought, heat shock, iron deficiency, and alkaline stress could change the period and phase of the expression of core clock genes in soybean (Li M. et al., 2019). Under alkaline stress, the leaf movement of wild soybean (*Glycine soja*) was roughly aligned with the clock gene expressions which showed an advancement of phase (Li M. et al., 2019). However, the leaf movements under drought and heat stress, and in iron-deficient plants, went out of sync with the core clock gene expressions, suggesting that different stresses may have different physiological effects (Li M. et al., 2019). Treatment of soybean with arsenate (As[V]) and arsenite (As[III]) could lead to changes in the amplitude of diurnal expressions of *GmLCL1*, *GmPRR9*, *GmELF4*, and *GmGI* (*Gigantea*), depending on the tissue and the form of arsenic compounds (Vezza et al., 2020). Stomatal movements and expressions of antioxidative enzymes were also altered by the As(V) and As(III) treatments. Yet, the link between the changes in core clock gene expressions and physiological changes remain elusive (Vezza et al., 2020). All in all, the direct physiological consequences of the alteration of the soybean circadian clock as a result of stresses are mostly uncharacterized.

During the domestication and improvement processes, breeders have selected for new soybean varieties with the aim to improve yield by mainly modifying the plant architecture and ensuring the soybean plant will reach maturity within a desirable timeframe, especially when adapting to a new cultivation region or habitat. It is known that artificial selection has enriched some minor alleles compared to the gene pool of the progenitor populations (Lam et al., 2010). Using the latest technologies in genomic studies, it is discovered that breeders, without prior knowledge of the circadian clock, have inadvertently introduced a few important and dominant modifications to the core clock components. The soybean genome has recently undergone two rounds of whole-genome duplication. Some of the clock component genes have diversified in functions after duplication, which further complicates the studies on their functions. Genetic manipulation of the circadian clock components of soybean using the well-studied Arabidopsis circadian clock model as a guide could help further improve soybean productivity. With the recent population genomic data, three circadian clock components, GmPRR3, GmELF3, and GmGIa from soybean were well-supported to be under artificial selection. In the following sections, we have summarized the recent discoveries in the modifications of these three circadian clock components in soybean during domestication.

## Modifications in Circadian Clock Component Genes in the Cultivated Soybean

### Mutations in a Pair of *Pseudo Response Regulator 3* (*PRR3*) Genes Resulted in Early Maturation

Earlier studies on the molecular controls of soybean maturation mainly focused on the *E* locus (Cober and Voldeng, 2001; Liu et al., 2008; Watanabe et al., 2009; Cober et al., 2010; Watanabe et al., 2011; Xia et al., 2012; Dissanayaka et al., 2016; Zhao et al., 2016; Lu et al., 2017; Samanfar et al., 2017; Yue et al., 2017). It was only recently that two major-effect quantitative trait loci (QTLs) on chromosomes 11 and 12 controlling maturation time and flowering time were found in wild soybeans or landraces (Qi et al., 2014; Lu et al., 2016, 2020; Fang et al., 2017; Pan et al., 2018; Li M. W. et al., 2019; Li C. et al., 2020; Ogiso-Tanaka et al., 2019). Dissection of these two QTLs identified two *Pseudo Response Regulator 3* genes, *GmPRR3a*/*Tof11* and *GmPRR3b*/*Tof12* on chromosomes 11 and 12, respectively, as the causal genes for controlling these two functions (Li M. W. et al., 2019; Li Y. et al., 2019; Li C. et al., 2020; Li Y. H. et al., 2020; Lu et al., 2020).

There are five *PRR* genes in Arabidopsis, including *PRR9*, *PRR7*, *PRR5*, *PRR3*, and *TOC1/PRR1*, which are all expressed in a circadian fashion and their expressions peak sequentially (Staiger et al., 2013; Gil and Park, 2019). These PRR proteins are typified with a pseudo receiver (PR) domain, an ERF-associated amphiphilic repression (EAR) motif, and a CONSTANTS, CO-like and TOC1 (CCT) domain (Gendron et al., 2012). Compared with other Arabidopsis PRR proteins, two non-synonymous substitutions naturally occurred on the EAR motif and CCT domain, respectively, of AtPRR3. These two substitutions are basically fixed in all *Arabidopsis thaliana* ecotype (Alonso-Blanco et al., 2016). These substitutions made AtPRR3 somehow behaves differently from other PRRs (Para et al., 2007). Unlike other PRRs which play direct roles in transcript repression, AtPRR3 probably participates in the circadian clock through stabilizing TOC1/PRR1 but without directly interacting with DNA through the CCT domain (Para et al., 2007). However, the soybean genome preserved functional alleles of PRR3 with intact EAR motif and CCT domain (Li C. et al., 2020; Lu et al., 2020), making soybean a good model for the study of this clock component.

A recent investigation of the growth period QTLs on chromosomes 11 (*Gp11*) and 12 (*Gp12*) using a cultivated-wild soybean recombinant inbred lines (RIL) population identified major-effect mutations in *GmPRR3a* and *GmPRR3b* (Li M. W. et al., 2019). A frameshift mutation was found in *GmPRR3a* (*GmPRR3a^*C*08^*) and a nonsense mutation in *GmPRR3b* in the cultivated parent C08 (*GmPRR3b^*C*08^*) by comparing the reference genome of the soybean cultivar Williams 82 to that of the wild soybean parent W05 (*GmPRR3a^*W*05^* and *GmPRR3b^*W*05^*) (Li M. W. et al., 2019). Both mutations bring about a pre-mature stop codon to the coding sequence and lead to the loss of the *C*-terminal CCT domain in the encoded proteins (Li M. W. et al., 2019). The end result was the shortening of the growth period of the soybean plant. Analysis of resequencing data suggested that the mutations are almost fixed in the improved cultivars, inferring that the mutations were strongly selected for and played important roles in domestication or during improvement (Li M. W. et al., 2019).

Genome-wide association mapping using 279 landraces discovered 16 flowering time and maturation time QTLs (Li C. et al., 2020). Linkage disequilibrium block analysis narrowed down the QTL on chromosome 12 to a single gene, *GmPRR3b*, confirming it to be the causal gene of the maturation and flowering time phenotypes (Li C. et al., 2020). In the same study, eight haplotypes of *GmPRR3b* were discovered (H1–H8). H1 has a stop-gain near the start codon (C43T), hence appearing to be a complete knockout of the *GmPRR3b* gene. H2, H3, and H5 have non-synonymous single-nucleotide polymorphisms (SNPs) outside of the conserved domains, which are assumed to be fully functional. H4 is the full-length *GmPRR3b* that is the same as *GmPRR3b^*W*05^* and is the dominant allele in wild soybean. H6 is the sole haplotype in the elite cultivar which has an ochre mutation and the loss of the CCT domain (equivalent to GmPRR3b^*C*08^). Both H7 and H8 have a non-synonymous mutation in the PR domain (Li C. et al., 2020). Studies on the flowering time of landraces carrying the H4–H8 haplotypes showed that alleles with mutations in the PR domain (H7 and H8) or with a truncated CCT domain (H6) have an early flowering phenotype (Li C. et al., 2020).

A more recent genome-wide association mapping using a panel of 424 soybean accessions also identified *GmPRR3a* and *GmPRR3b* in the QTLs *Tof11* and *Tof12* as the major causal genes controlling flowering and maturity (Lu et al., 2020). Molecular dating suggested that both alleles of *GmPRR3* in *tof11* and *tof12* lost their CCT domain ∼8,000 and ∼10,500 years ago, respectively (Lu et al., 2020). While only a tiny fraction of the *tof11* allele occurred alone, it is proposed that the *tof11* allele arose from the *tof12* background (Lu et al., 2020).

**FIGURE 1:**
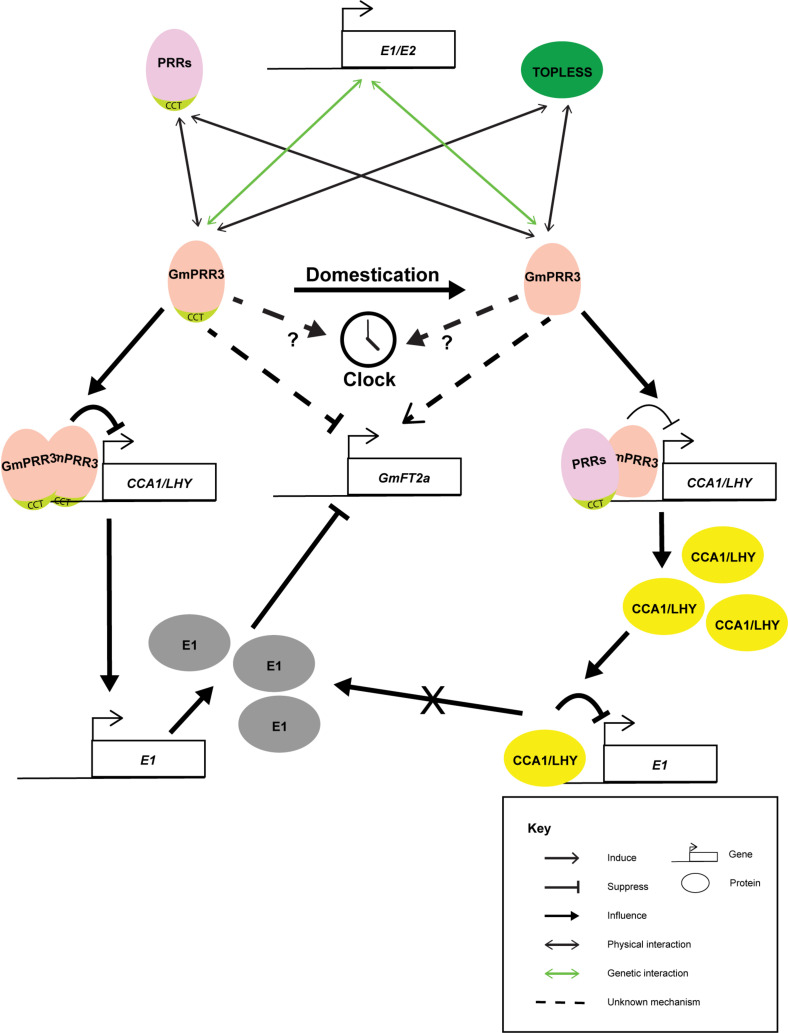
Cartoon summarizing the known interactions of GmPRR3a and GmPRR3b. *GmPRR3s* are genetically interacting with *E1* or *E2* loci in controlling flowering time and maturation of soybean probably through the regulation of *GmFT2a* expression. During domestication and soybean improvement, alleles of GmPRR3s without CCT domain were selected. GmPRR3s without CCT domain have weakened suppression of the expression of *CCA1*/*LHY* genes. The interaction of GmPRR3s without CCT domain may rely on other PRR proteins. Thus, the derepression of *CCA1*/*LHY* will in turn suppress the expression of *E1*. The suppression of *E1* maybe one of the routes that GmPRR3s without CCT domain derepress *GmFT2a*.

Based on the above studies, it is agreed that the domestication has selected for the alleles of *GmPRR3* without the CCT domain, leading to the early flowering phenotype and possibly altering the plant architecture related to yield (Figure 1) (Li M. W. et al., 2019; Li C. et al., 2020; Lu et al., 2020). GmPRR3s without the CCT domain probably promote flowering through derepressing the flower-promoting *Florigen T* gene, *GmFT2a*, under long-day conditions. It is interesting that a null allele with an 8.6% allele frequency in the wild soybean population was not selected (Li C. et al., 2020). This null allele was completely wiped out in the landrace (Li C. et al., 2020). It is highly possible that a complete loss of GmPRR3 functions may be detrimental. Knocking out the full-length *GmPRR3b* in the ZGDD background (equivalent to GmPRR3b^*H*4^) by CRISPR/cas9 led to a significant early flowering phenotype under long-day conditions (Wang L. et al., 2020). However, knocking out the *GmPRR3b^*C*08^*/*GmPRR3b^*H*6^*/*tof12* allele in the Jack and Tianlong backgrounds did not result in any significant change in flowering time (Wang L. et al., 2020) and a late-flowering phenotype with diminished yield components (Li C. et al., 2020), respectively. Furthermore, the overexpression of full-length *GmPRR3b* in Williams 82 generated a late-flowering phenotype (Lu et al., 2020), and the overexpression of *GmPRR3b^*C*08^*/*GmPRR3b^*H*6^*/*tof12* in Tianlong (Li C. et al., 2020) also produced a late-flowering phenotype and improved yield components. These observations suggest that the loss of the CCT domain in *GmPRR3* in domesticated soybean might have gained new functions for the protein that are related to yield and plant architect which is not present in the null allele.

The actual link between *GmPRR3* and flowering time is largely unknown. There are also some controversies with the existing models. Expressions of *GmPRR3a* and *GmPRR3b* cycle robustly in both long-day and short-day conditions (Li M. W. et al., 2019; Li C. et al., 2020) similar to the other *PRR* genes. The expressions peak at the middle of the day no matter whether it was a long-day (the 8^th^ hour in a 16/8 hour light/dark cycle) or a short-day regime (the 4^th^ hour in a 8/16 hour light/dark cycle) (Li M. W. et al., 2019; Li C. et al., 2020). The amplitude of oscillation seems to be slightly affected by the *E3* and *E4* loci (Lu et al., 2020). It has been shown that *GmPRR3* may be genetically interacting exclusively with either *E1* (Lu et al., 2020) or *E2* (Li M. W. et al., 2019), while transcriptomic network analyses suggested that *GmPPR3* may interact with both (Pan et al., 2018). Overexpressed *GmPRR3b^*H*6^*, the allele without a CCT domain, was shown to bind to the *GmCCA1a* promoter and dampen the expression of *GmCCA1a* (Li C. et al., 2020), suggesting that GmPRR3b is playing a direct role in the soybean circadian clock. However, the CCT domain was known to be essential for PRR proteins to directly interact with DNA (Gendron et al., 2012). Thus, the interaction of *GmPRR3b^*H*6^* with DNA may depend on its interactions with other PRR proteins or TOPLESS-like transcription factors (Li M. W. et al., 2019). On the other hand, the overexpression of *GmPRR3b^*H*4^*, the allele with a CCT domain, resulted in stronger suppression of *GmCCA1a* expression (Li C. et al., 2020), which could be the result of direct interactions with the *GmCCA1a* promoter. *Tof11* was also shown to be potentially able to bind to the promoters of *LHY/CCA1s* and suppress their expressions (Lu et al., 2020). Thus, Lu et al. (2020) proposed that GmPRR3 with a functional CCT domain suppresses *GmCCA1*/*LHY* expression, which leads to the derepression of *E1*. The high *E1* level in turn suppresses *GmFT* expressions, thus delaying flowering. Therefore, the mutated version of GmPRR3 without a CCT domain will lead to a higher expression of *GmCCA1*/*LHY* and the subsequent early flowering phenotype (Lu et al., 2020). However, this was soon questioned by the observation of Li et al. as the complete knockout of *GmPRR3b^*H*6^* had led to higher expressions of *GmCCA1a* and *GmFT2a* and a lower expression of *E1* (Li C. et al., 2020). Although this expression pattern is consistent with the assumption that GmPRR3b represses the expression of *LHY/CCA1s*, which act as repressors for *E1* (Lu et al., 2020), but the lower *E1* expression is not tally with the observed late-flowering phenotype (Li C. et al., 2020). Two possibilities on the delayed flowering were proposed. One is the involvement of pathway that is not mediated by CCA1/LHY-E1-FT2a module and the other is the consequence of alternated growth of *PRR3b* mutant (Li C. et al., 2020). The first possibility could be likely as E1 is a legume specific protein, while rice OsPRR37, a far homolog of GmPRR3, can influence the expression of *Hd3a*(*FT*) in the absent of E1 like protein (Matsubara et al., 2014). More delicate genetic studies will be needed to resolve these obstacles. Furthermore, studies suggested that *GmPRR3* mainly regulated the expression of *GmFT2a* but not *GmFT5a* (Li M. W. et al., 2019; Li C. et al., 2020), which resembled the regulation of *E2* (Lu et al., 2020). Nevertheless, *GmFT5a* may also be a target under certain circumstances. Therefore, further investigations into the interactions between *GmPRR3* and *GmFT5a* is required to solve this puzzle. Actually, the rice OsPRR37 can either promote or suppress flowering depending on the genetic background (Zhang et al., 2019). Thus, controversies of GmPRR3 functions in previous studies were due to the inadequate study of these genes.

### Live Long in the Tropics With Alterations in the *J* Locus

Soybean is a strict short-day species. Soybean plant normally requires staying vegetative for around 2 months to accumulate enough biomass to generate reasonable yield. Yet, a short-day photoperiod will trigger premature flowering of soybean plants and eventually result in low yield (Board and Hall, 1984). Thus, seasonal and latitudinal shifting of day-length have limited the growing season and acreage of soybean cultivation. However, according to model prediction, it is likely that more soybean will be grown in the tropics in 2100 (Fodor et al., 2017) where daylength is inductive for flowering in soybean.

Some late-flowering soybean cultivars in tropical regions were described as early as 1970s (Hartwig, 1970; Hartwig and Kiihl, 1979), especially in Brazil. These varieties could maintain a long juvenile (LJ) phenotype under short-day conditions, which enabled them to generate adequate seeds in the scale suitable for agricultural production (Destro et al., 2001; Carpentleri-Pipolo et al., 2002). The *E6* (Bonato and Vello, 1999) and *J* (Ray et al., 1995) loci were independently identified to control the LJ phenotype and were later found to be possibly closely linked genes (Cober, 2011; Li et al., 2017). Although there are other potential loci controlling the LJ phenotype (Destro et al., 2001; Carpentleri-Pipolo et al., 2002), the *J* locus is the best characterized.

Study of the *J* locus using a RIL population from a conventional juvenile variety, Zhonghuang 24, and an LJ variety, Huaxia 3, narrowed the causal gene to an indel (AT > A) in Glyma.04G050200 (Yue et al., 2017). Glyma.04G050200 (*GmELF3*) encodes a homolog of the Arabidopsis Early Flowering 3 (AtELF3). The deletion of the nucleotide (AT > A) results in truncation of the protein corresponding to the recessive *j* in the LJ variety (Yue et al., 2017). Constitutive expression of the full-length *GmELF3* promoted flowering in transgenic plants in the LJ parent, Huaxia 3, background (Yue et al., 2017). Another study identified a 10-bp deletion and a deletion of a cytosine in the *ELF3* of BR121 and PI 159925, respectively, to be the causal mutations of the LJ phenotype in these varieties (Lu et al., 2017).

**FIGURE 2:**
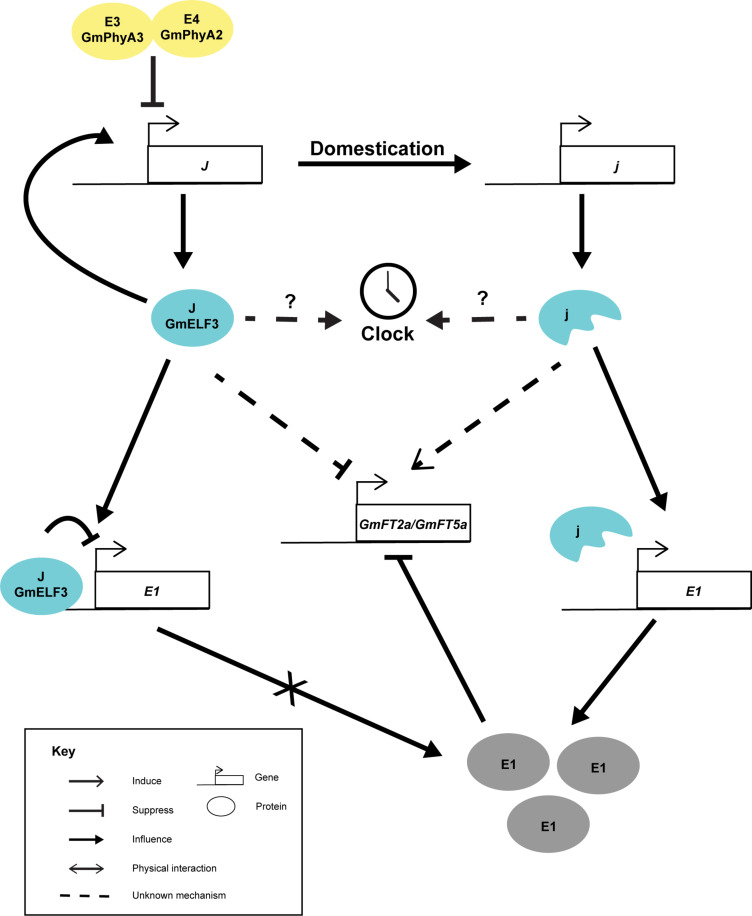
Cartoon summarizing the known interactions of *J*. The *J* locus possibly perceive light signal through *E3* and *E4* loci. The *J* locus encodes GmELF3 (J), which auto-regulates its own expression. GmELF3 can directly acts on *E1* promoter and suppress its expression. Without *E1* expression under short day conditions, *GmFT2a* and *GmFT5a* are highly induced and lead to unproductive flowering. In the opposite, domestication has selected *j* alleles in tropical regions. The j proteins are unable to auto-regulate its own expression and has no ability to bind to *E1*. Under this circumstance, E1 proteins are produced and suppresses the expression of *GmFT2a* and *GmFT5a* promoter under short day conditions which lead to the long juvenile phenotype.

*GmELF3* shows a circadian expression pattern and peaks at dusk (Lu et al., 2017), resembling the expression pattern of *AtELF3*. Interestingly, the loss-of-function allele of *GmELF3* did not oscillate diurnally, suggesting that an intact *GmELF3 per se* is essential to sustain its own expression pattern (Lu et al., 2017). Nevertheless, the effect of this *GmELF3* mutation on soybean circadian rhythm is not yet characterized. Like other *E* loci which are inter-dependent, the expression of *GmELF3* is slightly suppressed by *E3E4* (*GmPHYA3* and *GmPHYA2*) compared to the *e3e4* lines in the Harosoy background (Figure 2) (Lu et al., 2017). Yet, it is not known whether the higher expression of *GmELF3* in the *e3e4* background was due to the alteration in light input to the clock or due to a direct effect of the loss of GmPHYA functions. On the other hand, the functions of the *J* locus has been shown genetically to rely on *E1* (Lu et al., 2017). Only an intact GmELF3 and not the truncated protein can physically interact with the promoter of *E1*, probably as part of the evening complex (Lu et al., 2017). The recessive *j* allele was thought to suppress *FT* expression through *E1*, as the expression of *E1* was derepressed in the near-isogeneic line having a recessive *j* (NIL-*j*) with reference to NIL-*J* under inductive short-day conditions (Lu et al., 2017). Thus, although *j* allele was able to delay flowering in the *e1*^*as*^ (a weak allele of *E1*) background, the effect was less prominent than in the *E1* background (Lu et al., 2017). But still, it is possible that GmELF3 can alter flowering through alternative pathways. Taking rice Hd17(OsELF3) as an example. Hd17 mediates the expression of *OsPRR*s, *OsLHY*, *OsGI*, *Ghd7*, and *Hd1*(*CO*), which are acting in multiple pathways that affect *Hd3a*(*FT*) and flowering (Zhao et al., 2012; Yang et al., 2013; Matsubara et al., 2014).

Unlike other genes artificially selected, *J* is not widespread among soybean cultivars. Rather, *J* is tailored for soybean cultivation in tropical regions far away from the temperate origin of wild soybean (Lu et al., 2017). For instance, the Huaxia 3 allele (AT > A) of *J* can only be found in southern China (Yue et al., 2017) and eight other loss-of-function alleles (*j-1* to *j-8*) were all found in low-latitude regions (Lu et al., 2017). One of these alleles was the result of induced mutation (Lu et al., 2017), which unintentionally mutated a core clock gene.

There is trade-off for having the recessive *j* alleles. A recent study has found that near isogenic lines with a recessive *j* were more sensitive to salt stress compared to those having the dominant *J* (Cheng et al., 2020). Transient overexpression of *J* in soybean hairy roots produced composite plants with higher salt tolerance, as expected (Cheng et al., 2020). A transcriptomic study comparing NIL-*j* and NIL-*J* showed that NIL-*j* had significantly lower expressions of ∼95 stress-related transcription factor genes (Cheng et al., 2020). Higher expressions of *GmWRKY12*, *GmWRKY27*, *GmWRKY54*, *GmNAC11*, and *GmSIN1* were confirmed in *J*-overexpressing transgenic hairy roots (Cheng et al., 2020). It is not known whether *J* targeted the promoters of these genes directly, or if *J* altered the circadian clock, which led to different stress responses (Park et al., 2016; Coyne et al., 2019).

### Gigantea Is a Big Target for Modification During Domestication and Improvement

Gigantea (GI) is not directly involved in the circadian clock transcription feedback loop in the model plant, Arabidopsis. However, it has been found playing crucial roles in clock functions, seasonal flowering, and many other important biological processes (Mishra and Panigrahi, 2015). In brief, GI assisted the maturation and accumulation of ZEITLUPE (ZTL), an F-box E3 ligase that is responsible for the degradation of clock components including TOC1/PRR1, PRR5 and CCA1 HIKING EXPEDITION (CHE) (Cha et al., 2017; Lee et al., 2019), so as to derepress *LHY*/*CCA1* and bring about the progression of the circadian rhythm. On the other hand, under inductive long-day conditions, the diurnal expression of Arabidopsis *GI* coincides with the circadian-controlled *FLAVIN-BINDING, KELCH REPEAT, F-BOX 1* (*FKF1*) (Mishra and Panigrahi, 2015). The GI-FKF1 thus is able to form a complex to degrade the flowering suppressors, cycling DOF factors (CDFs), allowing the transcription of *CONSTANTS* (*CO*). CO will then promote the expression of *FT* and result in flowering (Mishra and Panigrahi, 2015).

There are three copies of *GI* in the soybean genome, *GmGIa*, *GmGIb*, and *GmGIc*. *GmGIa* (Glyma.10g221500) was known as *E2* (Watanabe et al., 2011), which played major roles in the flowering and maturation of soybean (Wang Y. et al., 2016). The nucleotide diversity of *GIb* and *GIc* were not different between wild and cultivated soybeans (Wang Y. et al., 2016), suggesting that they were not targets for human selection. Analyses of 104 wild soybeans and 203 Chinese landraces identified 47 haplotypes (H1–H47) of *GmGIa* in total. Due to artificial selection, there were only three haplotypes (H1–H3) of *GmGIa* in the 203 landraces. Furthermore, the *GmGIa* in cultivated soybeans retained only 4.7% nucleotide diversity and 2.9% nucleotide polymorphism compared to 66% nucleotide diversity and 49% nucleotide polymorphism of the entire genome, respectively (Hyten et al., 2006; Wang Y. et al., 2016), implying a strong bottleneck in this gene.

H2 and H3 encode the full-length, 1,177 (or 1,170; Watanabe et al., 2011) amino acids of the GmGIa protein and are different in a single amino acid substitution from V_220_ to I_220_ (Wang Y. et al., 2016). The substitution appeared to have no significant effect on the GI function on flowering. H1 (*e2*) has a 66.95% frequency in the Chinese landraces compared to 4.81% in wild soybeans. Due to a premature stop codon in the 10^th^ exon, H1 encodes a protein with only 527 (or 521; Watanabe et al., 2011) amino acids and contributes to the early flowering and maturation phenotype (Wang Y. et al., 2016). So far, no Korean or Japanese wild soybean bearing the H1 haplotype has been found (Wang Y. et al., 2016). Therefore, it is proposed that H1 (*e2*) originated from China and was later introduced into the other East Asian regions (Kim et al., 2018). On the other hand, another haplotype with a premature stop codon in the 2^*nd*^ exon has been specifically described in Korean early flowering varieties. This suggests the mutation arose independently and only spread locally (Kim et al., 2018).

**FIGURE 3:**
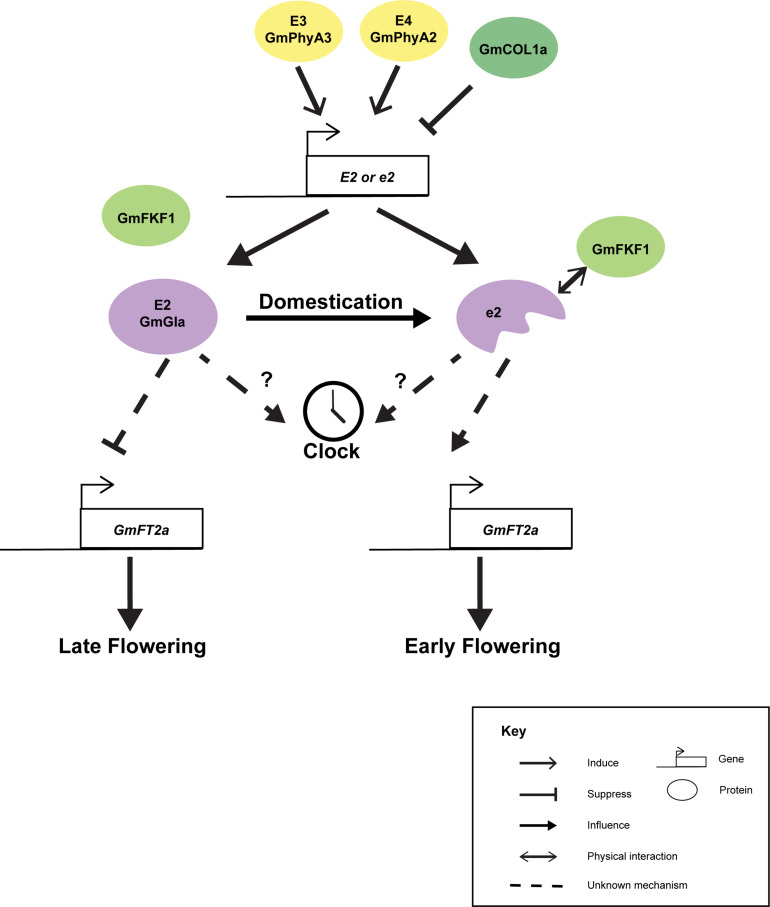
Cartoon summarizing the known interactions of *GmGIa*. Expression of *GmGIa* (*E2*) is controlled by *E3*, *E4* loci and GmCOL1a. During domestication, a truncated *GmGIa* gene (*e2*) was selected. The truncated protein but not the full-length protein can interact with GmFKF1 in yeast 2 hybrid assay. The full-length GmGIa (E2) protein can suppress the expression of *GmFT2a* and leading late flowering phenotype. In the opposite, *GmFT2a* is derepressed in *e2* background allowing early flowering phenotype.

Although *GmGIa* is well known as a gene controlling flowering and maturity, with the discrepancies between the short-day soybean and the long-day Arabidopsis, the actual molecular mechanism of how GmGIa regulates flowering and maturation is largely unknown. It is believed that the function of GmGIa has diverged from the GIs in other plant species. For instance, the full-length GmGIa serves as a flowering suppressor while its Arabidopsis counterpart acts as a flowering promoter (Watanabe et al., 2011). Interestingly, full-length OsGI from rice, a short-day monocot, serves as flowering suppressor (Hayama et al., 2003). Seemingly, GI functions in long-day and short-day plants are different. The expression of *GmGIa* was found to be correlated with the expression of *GmFT2a* but not that of *GmFT5a* under natural light conditions (Figure 3) (Watanabe et al., 2011). Yet, the mechanistic link between *GmGIa* and *GmFT2a* expressions is still missing. The expression of *GmGIa* is suppressed in the *e3e4* background or by the overexpression of *GmCOL1a* (Cao et al., 2015). A combination of *E2* and *E3* alleles can synergistically improve seed yield in July sowing in Japan (Kawasaki et al., 2018). Under long-day conditions, the expression of *GmGIa* oscillates following a circadian rhythm but is slightly out of sync with the expressions of the two *GmFKF1* genes (Li et al., 2013), while under short-day conditions, the expression of *GmGIa* is dampened and no longer oscillates (Li et al., 2013). This is something different from the expression of *AtGI* (Sawa et al., 2008) and *OsGI* (Hayama et al., 2003). Thus, the GI-FKF1 coincident model in Arabidopsis cannot be directly applied to soybean. Ectopic expressions of H2 and H3 in Arabidopsis delayed flowering while only the constitutive expression of H1 (a truncated GmGIa), but not those of H2 and H3, can rescue the early flowering phenotype of the *gi-2* Arabidopsis mutant (Wang Y. et al., 2016). This may be explained by the fact that GmFKF1 could only interact with the *N*-terminus but not the full-length GmGIa in yeast two-hybrid experiments (Li et al., 2013). It is possible that the Arabidopsis FKF1 can only be stabilized by the truncated but not the full-length GmGIa. Thus, ectopic expressions of H2 and H3 could not rescue the late-flowering phenotype of *gi-2* (Wang Y. et al., 2016). Furthermore, GmGIa has also been found to mediate the level of *miR172a*, which in turn regulates the stability of an *AP2*/*TOE1* mRNA that affects flowering time (Wang T. et al., 2016).

Mutations in *GI* in other species have been shown to affect salt stress tolerance, oxidative stress tolerance, and water use efficiency (Kurepa et al., 1998; Kim et al., 2018; Simon et al., 2020), but it is still largely unknown whether the alteration of *GmGIa* during domestication has also contributed to stress-related phenotypes in addition to the well-known effects on flowering and maturation.

## Discussion

The modifications of *GmPRR3*, *GmELF3*, and *GmGIa* as a result of artificial selection seem to complement one another. The truncated alleles of *GmPRR3a* and *GmPRR3b* are widespread in landraces and are almost fixed in improved cultivars. This may be due to the possibility that the alteration of *GmPRR3* confers an overall advantage in growth period as well as yield. On the other hand, the combination of different alleles of *E2* with other *E* loci partially define the maturity groups of soybean cultivars. Maturity groups are useful for selecting the appropriate cultivars at different latitudes to maximize yield. However, the effective application of *E2* is confined to the temperate and subtropical zones. In the tropics, *GmELF3* serves a similar function to *E2* for selecting the best adapted cultivars for that zone.

At the molecular level, the modifications of *GmPPR3* and *GmGIa* are interesting processes. It involved more than simply altering the gene expressions or completely knocking out the gene functions because knockout alleles also exist in wild accessions. Instead, domestication has introduced new functions to these two proteins. Mutation by deletion of the CCT domain of both copies of GmPRR3 would not have happened purely by chance, since they showed a certain degree of redundancy, only modification of both could maximize the effect (Li M. W. et al., 2019). Similar mutations or deletions of the CCT domain in *PRR37* alleles have also occurred during rice and sorghum domestication (Murphy et al., 2011; Koo et al., 2013). The plants having these CCT domain deletions in GmPRR3 behave differently from those complete *GmPRR3*-knockout lines (Li C. et al., 2020; Wang L. et al., 2020). The loss of the CCT domain abolished the DNA-binding ability of GmPPR3 but retained its protein–protein interaction capability. In this case, the truncated GmPRR3 may serve as an inhibitor in protein complexes and alter their functions. In the case of GmGIa, the truncation of the protein allows its interaction with GmFKF1. Interestingly, the morphologies of different Arabidopsis *gi* mutants differ drastically, implying that different parts of the GI protein are involved in diverse biological functions, and mutations in different parts of this protein could thus lead to quite different phenotypes (Mishra and Panigrahi, 2015). Based on this information, strategic modifications of *GmGIa* by genome editing may result in other possible changes to soybean as what researchers have done on the rice *waxy* gene (Chen and Mas, 2019; Huang et al., 2020). Although we have gained some knowledge of the soybean circadian clock components, in that they have crucial impacts on growth and development, the mechanisms of their actual involvement in the clock is still largely unknown. While the clock outputs are involved in diverse biological processes, ignorance of the mechanisms of how the clock components function while conducting genetic manipulation of the crop plant may result in undesired outcomes such as the alteration of metabolic profiles and stress responses. Besides, so far, there are many more circadian clock components identified in the soybean genome than the three discussed in this review (Liu et al., 2009; Liew et al., 2017; Marcolino-Gomes et al., 2017; Li M. et al., 2019). For example, in additional to GmPRR3a and GmPRR3b, there are 12 PRR homolog encoding genes in the soybean genome (Li M. W. et al., 2019). Nonetheless, their roles in soybean domestication and improvement are either less prominent or the genes have experienced less significant modification.

Modification of the circadian clock during domestication and improvement was entirely serendipitous. Selection was usually based on phenotypes at macroscopic levels. Thus, targets for modification are limited to those causing obvious phenotypic changes. However, with advanced knowledge in the circadian clock, soybean genomics, and genome editing technology, a second wave of modifications of the circadian clock components at the molecular level has become possible.

For example, isoflavones, a sub-group of flavonoids, are secondary metabolites unique to legumes and are beneficial to human either as food or nutraceuticals. Contrary to the model plant Arabidopsis and many other crop species, soybean and other legumes are able to fix nitrogen through a symbiotic relationship with rhizobia. The initiation of the host-symbiont interaction requires flavonoid signaling. Precursors of flavonoids are produced through the phenylpropanoid pathway, which is heavily regulated by the circadian clock at the transcriptional level (Harmer et al., 2000). Thus, well-designed genetic alterations of the circadian clock components could improve the production of these compounds, which raises the commercial values and also the nitrogen-fixing ability of soybean.

Although photosynthesis is dependent on daylength, light intensity, and temperature, the utilization of photosynthates at night is largely controlled by the clock (Graf and Smith, 2011). Since nitrogen fixation relies heavily on the availability of photosynthates supplied by the plant host, the ability of the microsymbiont to fix nitrogen could be impacted by the alteration in the circadian clock of the plant. At present, this is a completely unexplored area.

In addition to spreading soybean cultivation to lower latitudes, agriculture is also moving indoors. Advanced agricultural systems such as vertical farms or hydroponics are gaining popularity in more urban areas. Synchronization of the artificial light-dark cycle with the internal clock of the crop would be beneficial for maximizing crop production using these systems (Belbin et al., 2019). To achieve this goal, more in-depth knowledge of the circadian clock in soybean and other crop plants would be desirable.

In the past decade, since the official release of the first soybean genome assembly in 2010, identification of variants of soybean circadian clock components heavily relied on genome resequencing based on a single reference genome. Such strategy has limited power in the discovery of new genes and structural variations. With the advance of soybean genomics, more and more high-quality soybean genome assemblies are available (Shen et al., 2018; Valliyodan et al., 2019; Xie et al., 2019). A high-quality pan-genome of wild and cultivated soybeans are also reported recently (Liu et al., 2020). These genomes allow direct comparison of sequence of clock components from different soybean accessions which resulted in more accurate discovery of variants. Furthermore, better reference genomes also facilitate epigenetic study. It has been demonstrated that histone modifications have played crucial roles in regulating the core clock components in plant (reviewed in Chen and Mas, 2019; Du et al., 2019). Nevertheless, information of the roles of epigenetic on soybean domestication and soybean circadian clock function is scarce. Thus, exploration of the modification of circadian clock components and their regulation at epigenetic level could be the next chapter of soybean research.

## Author Contributions

M-WL drafted the manuscript. M-WL and H-ML edited and proofread the final manuscript. Both authors contributed to the article and approved the submitted version.

## Conflict of Interest

The authors declare that the research was conducted in the absence of any commercial or financial relationships that could be construed as a potential conflict of interest.
